# The Real-World Characteristics of Gender-Affirming Hormonal Use Among Transgender People in Thailand

**DOI:** 10.1016/j.esxm.2022.100513

**Published:** 2022-04-12

**Authors:** Sakditat Ittiphisit, Salin Amponnavarat, Natnicha Manaboriboon, Sira Korpaisarn

**Affiliations:** 1 Faculty of Medicine Vajira Hospital, Navamindradhiraj University, Bangkok, Thailand; 2 Department of Medicine, Faculty of Medicine Ramathibodi Hospital, Mahidol University, Bangkok, Thailand; 3 Faculty of Medicine Siriraj Hospital, Mahidol University, Bangkok, Thailand

**Keywords:** Transgender, Gender-Affirming Hormonal Therapy, Gender Dysphoria

## Abstract

**Background:**

Most Thai transgender people (TG) do not use gender-affirming hormone therapy (GAHT) under medical supervision.

**Aim:**

To understand the current real-world characteristics of GAHT among TG.

**Methods:**

A cross-sectional survey was conducted using an online questionnaire between September and December 2020. TG, who resided in Thailand for more than 1 year, were included. Self-reported demographic data and characteristics of GAHT were obtained. The reported GAHT were compared to the reference regimen recommended by the 2017 Endocrine Society Clinical Practice Guideline.

**Outcomes:**

The characteristics of GAHT used among TG and factors associated with hormonal use outside the reference regimen were reported.

**Results:**

A total of 401 TG were included in the analysis. Of these, 249 (62%) were transgender men (TM). Most TM (81%) and transgender women (88%) were using GAHT. Only 297 TG provided a complete hormone regimen. A total of 224 TG (75%) used GAHT outside the reference regimen. The main reasons in TM were using intramuscular testosterone with a higher dose per injection and less frequent intervals. In transgender women, using oral contraceptive pills and cyproterone acetate 12.5 mg/d were the two most common reasons. A univariate analysis revealed factors associated with hormonal use outside the reference regimen, including age at a survey participation (OR 1.04, 95%CI 1.00–1.08, *P* = .047), age at hormone initiation (OR 1.04, 95%CI 1.01–1.08, *P* = .015), TM (OR 2.08, 95%CI 1.22–3.56, *P* = .007) and using GAHT, not under medical supervision (OR 1.78, 95%CI 1.04–3.05, *P* = .037). The multivariate analysis showed that only living outside the capital city was solely statistically significant (AOR 1.77, 95%CI 1.02–3.05, *P* = .041).

**Clinical Implications:**

Enhancing health literacy in GAHT among TG is crucial, especially TG not living in the capital city.

**Strengths and Limitations:**

This study demonstrates a current real-world practice of GAHT use among TG, both under and not under medical care. However, the causality could not be concluded due to the nature of the cross-sectional observation study, and results come with a recall bias.

**Conclusion:**

There is a high prevalence of GAHT use outside the reference regimen. The only factor associated with hormonal use outside the reference regimen is living outside the capital city.

**Ittiphisit S, Amponnavarat S, Manaboriboon N, et al. The Real-World Characteristics of Gender-Affirming Hormonal Use Among Transgender People in Thailand. Sex Med 2022;10:100513.**

## INTRODUCTION

Transgender people (TG) are individuals who have their gender identity different from their birth-assigned sex. A global meta-prevalence estimate of the number of TG who sought or received gender affirmation therapy was reported as 9.2 per 100,000 population with a range between 0.9 and 35.0.^1^ Although there is no official registration of TG in Thailand, several old reports revealed a wide range of prevalence from 1:180 to 1:3000.^2^, ^3^, ^4^ In addition, Thai demographic data in 2006 showed that a majority of TG (69.5%) resided in Bangkok, the capital city of Thailand.^5^

Most TG in Thailand use gender-affirming hormone therapy (GAHT), and up to 94% of transgender women (TW) use feminizing hormones.^5^ Recommended feminizing hormone regimens comprise (1) estrogen- to promote female secondary sexual characteristics, and (2) androgen-lowering drugs- to inhibit male secondary sexual characteristics. Common estrogen routes of administration are oral, transdermal, and intramuscularly parenteral. In addition, androgen-lowering drugs, including cyproterone acetate, spironolactone, and gonadotropin-releasing hormone (GnRH) agonists, are parts of feminizing hormone regimens. The preference of each medication varies depending on underlying medical conditions, cost, availability, and physicians’ choice.^6^ Among transgender men (TM), testosterone is a crucial hormone to stimulate male secondary sexual characteristics and induce virilization. The standard routes of administration include parenteral (intramuscular and subcutaneous), oral, transdermal, transbuccal, and implantable. Both TW and TM need to continue GAHT to maintain the desired sexual characteristics while the age of discontinuation or de-intensification of the treatment remains unclear.^6^

Hormonal use under medical supervision yields a benefit in terms of clinical outcomes.^6^^,^^7^ A study in 2011 in Thailand revealed that 89% of TW self-purchased hormones from the pharmacy. In contrast, only 31% and 5% received them from clinics and hospitals, respectively.^8^ Another study done in 2013 revealed that Thai TG mainly used hormone therapy through recommendations from friends or acquaintances.^9^ TG in several developing countries can purchase hormones over the counter through a pharmacy or an illegal dealer. This practice is not monitored and could lead to complications from GAHT, for example, venous thromboembolism, myocardial infarction, stroke, in TW, and polycythemia in TM.^10^, ^11^, ^12^ However, these reports were almost a decade ago, while knowledge in transgender medicine has recently expanded. This study aims to identify the current characteristics of GAHT among TG and reveal the factors associated with hormonal use outside the reference regimen. We hypothesize that although transgender care has been improved, the real-world practice in the TG community may be the same and thus requires attention in order to see improvement.

## MATERIALS AND METHODS

The present study has been carried out in accordance with The Code of Ethics of the World Medical Association (Declaration of Helsinki) for experiments involving human subjects. It was approved by the Institutional Review Board of the study site, and the written informed consents were exempted. This study was a cross-sectional survey study through an online questionnaire. The questionnaires were distributed between September and December 2020 via TG-related online social media platforms and LGBTQ+ alliances. The inclusion criteria were (i) individuals who identified themselves as a TG defined by a disparity between their birth-assigned sex and their gender identity and (ii) individuals who have resided in Thailand for at least 1 year. The eligibility was asked in the first question and self-screened by participants. Then participants provided consent to continue the survey if they were qualified. The questionnaire specifically asked for only participants who identified themselves as transgender people to be included, therefore other populations apart from transgender people were excluded. We expected more than 384 participants given that the exact transgender population is not known with the absolute error of 5% and at type 1 error of 5%.

The online survey was divided into 3 sections: personal information, gender history, and information regarding hormonal use. To ensure anonymity, personal information did not include the name or any identifiable variables. Personal information included current age, area of residence, socio-economic backgrounds, education, and occupation. Gender history included gender identity, age of gender identity realization, birth-assigned sex, gender expression, sexual orientation, and history of gender-affirming surgery. The first 2 parts were adopted from a general intake form used for an initial visit at a transgender clinic at our institute. Finally, the hormonal information comprised the current hormonal regimen, age of hormone initiation, sources of hormonal treatment, sources of knowledge regarding hormonal use, and whether hormonal use was supervised by a healthcare professional or not. The final part was developed by providers prescribing GAHT at a transgender clinic at our institute. The survey was assessed by non-medical transgender people and adjusted accordingly to ensure that the language was sensitive and appropriate.

### The Reference Hormone Regimen

At our institute, GAHT recommended by the 2017 Endocrine Treatment of Gender-Dysphoric/Gender-Incongruent Persons: An Endocrine Society Clinical Practice Guideline,^11^ was used as a standard regimen. Data with regard to the self-reported hormonal regimen, including the names of medication, doses, and intervals, were compared to a reference regimen. The hormone regimens, which did not follow the 2017 Endocrine Society Guidelines, were further grouped as “hormonal use outside the reference regimen”. Those compatible with the guidelines were classified as “hormonal use in accordance with the reference regimen”. To be considered as the latter, all names of medication, doses, and intervals needed to align with the reference regimens. Only provided regimens with the complete name of hormones, doses, and intervals were included for analysis.

In Thailand, all medications recommended by the 2017 Endocrine Society Guidelines are available, except for oral 17-beta estradiol. An oral estradiol valerate and oral estradiol hemihydrate, the esterified form of 17-beta estradiol, are used as a standard feminizing hormone. These 2 medications are converted to 17-beta estradiol once administered into the body system.

### Statistical Analysis

Data were analyzed using STATA version 16.0 (StataCorp. 2019. Stata Statistical Software: Release 16. College Station, TX, USA: StataCorp LLC.). Descriptive data are expressed as counts (%) for categorical variables and mean with ±SD for numerical variables. T-test was used to compare continuous variables between two groups. Chi-square test was used to compare categorical variables between two groups, and Fisher's exact test was used where appropriate. We used logistic regression to determine the relationship between socio-demographic data and hormonal use outside the reference regimen. In the univariate analysis, odds ratio (OR) were presented for each variable, including age (per year), age of gender identity realization (per year), age of hormone initiation (per year), gender (transgender woman or transgender man), area of residence, educational level, employment status, prior gender-affirming surgery, sources of knowledge for hormonal use, and whether their use was under medical supervision, with the 95% confidence interval (CI) and *P* values. Variables with *P* < .10 in the univariate analysis were further included in the multivariate analysis using multiple logistic regression, and adjusted odds ratio (AOR) was reported for each included parameter. A *P* < .05 was considered statistically significant.

## RESULTS

Four hundred thirty-two subjects responded to the surveys, of which 401 were eligible and included in the final analysis. Of these, 249 (62%) self-identified as TM, while 152 (38%) self-identified as TW. Table 1 shows demographic data and GAHT history. The mean age of TW is 25 ± 7 years, which is younger than TM, 30 ± 7 years. TW started GAHT at age 17 ± 4.79 years, while TM started GAHT significantly later at age 28 ± 7.67 years (*P* < .001). Twenty-seven percent of TM and 52% of TW received an education lower than a bachelor's degree (*P* < .001). Half of TW (53%) were unemployed. Most TW (80%) did not undergo gender-affirming surgery, while almost half of the TM (48%) had gender-affirming surgery done.

**Table 1:** Demographic characteristics of the 401 transgender people

	Transgender man	Transgender woman	*P*-value
Number (%)	249 (62.1)	152 (37.9)	
Age (years), mean ± SD	30 (±7.3)	25 (±7.3)	<.001
Age of gender identity realization (years), mean ± SD	7.8 (±4)	8.0 (±4.5)	.625
Age of hormone initiation (years), mean ± SD	28.0 (±7.7)	17.5 (±4.8)	<.001
Area of residence, number (%)			
Bangkok	108 (43.4)	53 (34.9)	.152
Outside Bangkok	141 (56.6)	99 (65.1)	
Education level, number (%)			
Less than bachelor's degrees	67 (26.9)	79 (52.0)	<.001
Bachelor's degrees or higher	182 (73.1)	73 (48.0)	
Employment status, number (%)			
Employed	205 (82.3)	72 (47.37)	<.001
Unemployed	44 (17.7)	80 (52.63)	
Gender-affirming surgery, number (%)			
Never	131 (52.6)	122 (80.3)	<.001
Orchiectomy/removal of the testis	N/A	11 (7.2)	
Hysterectomy/removal of ovaries	29 (11.7)	N/A	
Other surgeries	89 (35.7)	19 (12.5)	
Gender-affirming hormone therapy, number (%)			
Currently use gender-affirming hormones	202 (81.1)	133 (87.5)	.098
Former users/Never	47 (18.9)	19 (12.5)	
Sources of hormone knowledge, number (%)	202 (100)	133 (100)	
Internet	128 (63.4)	71 (53.4)	<.001
Social media	9 (4.5)	14 (10.5)	
Friends or family	10 (5.0)	24 (18.0)	
Hospital/clinic	52 (25.7)	24 (18.0)	
Advertisements	2 (1.0)	N/A	
Others	1 (0.5)	N/A	
Under medical supervision, number (%)			
Yes	186 (92.1)	24 (18.0)	<.001
No	16 (7.9)	109 (82.0)	

N/A = not applicable; SD = standard deviation.

Most TM (81%) and TW (88%) were currently using GAHT at the time of the survey. The most common source of hormone information was from the internet in both TM (51%) and TW (47%). The second most common source of information was from clinics/hospitals reported in 21% of TM and 36% of TW. Only 18% of TW, in contrast with most TM (92%), reported using GAHT under medical supervision (*P* < .001). Feminizing and masculinizing hormone regimens are shown in Table 2. Most TW used oral estradiol valerate (57%), a prodrug of 17beta-estradiol, or oral cyproterone acetate (54%). However, up to 21% used oral contraceptive pills. Estrogen gel and injectable estrogen were reported in 12% and 11%, respectively. Surprisingly, 17% of TW simultaneously used three or more medications (11% for three drugs, 4% for four drugs, and 2% for five drugs). Regarding masculinizing hormone regimen, most TM (89%) used testosterone enanthate injection followed by testosterone undecanoate injection (7%). Only 1 TM reported using testosterone gel, while none used oral testosterone. All TM used only one medication for masculinization.

**Table 2:** Feminizing and masculinizing medications reported by transgender people

Feminizing hormone	Number (%)	Masculinizing hormone	Number (%)
Oral estradiol valerate	76 (57.1)	Testosterone enanthate injection	180 (89.1)
Oral cyproterone acetate	73 (54.9)	Testosterone undecanoate injection	15 (7.4)
Oral contraceptive pills	28 (21.1)	Testosterone isocaproate injection	1 (0.5)
Oral estradiol	19 (14.3)	Testosterone gel	1 (0.5)
Estrogen gel	16 (12.0)	Growth hormone	1 (0.5)
Estrogen/estradiol Injection	15 (11.3)		
Oral phytoestrogen	2 (1.5)		
Hydroxyprogesterone caproate Injection	2 (1.5)		
Pueraria mirifica (Kwao krua)	1 (0.8)		
Estrogen patch	1 (0.8)		
Oral progesterone	1 (0.8)		
Spironolactone	1 (0.8)		
Total	235	Total	198
Number of medications in feminizing regimen		Number of medications in masculinizing regimen	
1 medication	62 (46.6)	1 medication	198 (100)
2 medications	49 (36.8)	2 medications	0
3 medications or more	22 (16.5)	3 medications or more	0

GAHT were reported in 202 TM and 127 TW, of which 186 TM and 111 TW provided complete regimens, including names, doses, and intervals. According to the recommended regimens in the 2017 Endocrine Society Guidelines, 81% of TM and 67% of TW used GAHT outside the reference regimen (Table 3), with a total number of 224 TG (75%). For TM, the main reason for hormonal use outside the reference regimen was using doses other than the recommended doses (57%). All used more than 200 mg of testosterone enanthate per injection. Using intervals apart from the recommended intervals was reported in 41% of TM. Almost all were less frequent intervals. Only 3 TM (2%) used different hormones from the reference medication. In TW, 47% used doses apart from recommended doses, and 49% used drugs other than recommended medications. Three TW (4%) reported using solely anti-androgen as a feminizing regimen. The characteristics of GAHT outside the reference regimen are shown in Table 4.

**Table 3:** Characteristics of transgender people stratified by hormonal use outside the reference regimen and hormonal use in accordance with the reference regimen

	In accordance with the reference regimen	Outside the reference regimen
Number	73	224
Age (years), mean ± SD	26.85 (±8.27)	28.95 (±7.57)
Age of gender identity realization (years), mean ± SD	7.41 (±4.14)	7.76 (±4.14)
Age of hormone initiation (years), mean ± SD	22.08 (±8.55)	24.90 (±8.39)
Gender, number (%)		
Transgender man (n = 186)	36 (49.32)	150 (66.96)
Transgender woman (n = 111)	37 (50.68)	74 (33.04)
Area of residence, number (%)		
Bangkok	36 (49.32)	84 (37.50)
Outside Bangkok	37 (50.68)	140 (62.50)
Education level, number (%)		
Less than bachelor's degrees	50 (68.49)	149 (66.52)
Bachelor's degrees or higher	23 (31.51)	75 (33.48)
Employment status, number (%)		
Employed	47 (64.38)	165 (73.66)
Unemployed	26 (35.62)	59 (26.34)
Gender-affirming surgery, number (%)		
Never	47 (64.38)	127 (56.70)
Had surgery	26 (35.62)	97 (43.30)
Orchiectomy	4 (5.48)	3 (1.34)
Hysterectomy and ovariectomy	6 (8.22)	20 (8.93)
Other surgeries	16 (21.92)	74 (33.04)
Sources of hormone knowledge, number (%)		
Internet	39 (53.42)	135 (60.27)
Social media	9 (12.33)	10 (4.46)
Friends or family	6 (8.22)	25 (11.16)
Hospital/clinic	19 (26.03)	52 (23.21)
Advertisements		1 (0.45)
Others		1 (0.45)
Under medical supervision, number (%)		
Yes	33 (45.21)	71 (31.70)
No	40 (54.79)	153 (68.30)

SD = standard deviation.

**Table 4:** Characteristics of GAHT outside the reference regimen (n = 224)

Feminizing hormone	n
Drug not in accordance with the reference regimen	39
Oral contraceptive pills	19
Combine >2 forms of estrogen	16
Use only CPA	1
Oral phytoestrogen	3
Dose and frequency not in accordance with the reference regimen	35
CPA 12.5 mg daily	19
CPA 6.25 mg daily	1
CPA 100 mg daily	3
Estradiol dose >6 mg/d (8–12 mg/d)	5
Estradiol dose <2 mg/d (0.5–1 mg/d)	3
Not taking medication daily	3
Estradiol gel 6 mg/d	1
Total	74
Masculinizing hormone	
Dose not in accordance with the reference regimen	86
Testosterone enanthate 250 mg per injection	83
Testosterone enanthate 225 mg per injection	2
Testosterone enanthate 300 mg per injection	1
Interval not in accordance with the reference regimen	61
Less frequent interval	59
Testosterone enanthate 200 mg every 3 wk	15
Testosterone enanthate 200 mg every 4 wk	6
Testosterone enanthate 175 mg every 3 wk	1
Testosterone enanthate 175 mg every 4 wk	1
Testosterone enanthate 150 mg every 3 wk	4
Testosterone enanthate 150 mg every 4 wk	5
Testosterone enanthate 125 mg every 3 wk	5
Testosterone enanthate 125 mg every 4 wk	5
Testosterone enanthate 100 mg every 3 wk	6
Testosterone enanthate 100 mg every 4 wk	3
Testosterone enanthate 75 mg every 2 wk	1
Testosterone enanthate 75 mg every 3 wk	1
Testosterone enanthate 50 mg every 2 wk	6
More frequent interval	2
Testosterone enanthate 150 mg weekly	1
Testosterone enanthate 125 mg weekly	1
Drug not in accordance with the reference regimen	3
Total	150

CPA = cyproterone acetate; GAHT = Gender-affirming hormone therapy.

Results of the univariate analysis under a simple logistic regression revealed several factors statistically significantly associated with a higher risk of hormonal use outside the reference regimen (Table 5), including age at the time of participating in the survey, for each year older (OR 1.04, 95%CI 1.00–1.08, *P* = .047), age of hormone initiation, for each year older (OR 1.04, 95%CI 1.01–1.08, *P* = .015), being a transgender man (OR 2.08, 95%CI 1.22–3.56, *P* = .007) and using GAHT, not under medical supervision (OR 1.78, 95%CI 1.04–3.05, *P* = .037). In addition, TG living outside Bangkok, the capital city of Thailand, showed a trend of higher risk of hormonal use outside the reference regimen (OR 1.62, 95%CI 0.95–2.76, *P* = .075). The multivariate analysis using multiple logistic regression is shown in Table 5. Included parameters for analysis were age at the time of participating in the survey, age of hormone initiation, gender, area of residence, and whether using GAHT under medical supervision or not. Only the area of residence remained statistically significant. Living outside Bangkok was associated with a higher risk of hormonal use outside the reference regimen (AOR 1.77, 95%CI 1.02–3.05, *P* = .041).

**Table 5:** Univariate and multivariate analysis of factors associated with hormonal use outside the reference regimen

	Univariate analysis		Multivariate analysis	
	OR (95%CI)	*P*-value	AOR (95%CI)	*P*-value
Age (per year)	1.04 (1.00–1.08)	.047*	1.01 (0.96–1.07)	.622
Age of gender identity realization (per year)	1.02 (0.96–1.09)	.53		
Age of hormone initiation (per year)	1.04 (1.01–1.08)	.015*	1.01 (0.96–1.07)	.639
Gender				
Transgender woman	(r)			
Transgender man	2.08 (1.22–3.56)	.007*	1.56 (0.65–3.75)	.317
Area of residence				
Bangkok	(r)			
Outside Bangkok	1.62 (0.95–2.76)	.075	1.77 (1.02–3.05)	.041*
Education level				
Bachelor's degrees or higher	(r)			
Less than bachelor's degrees	1.09 (0.62–1.93)	.755		
Employment status				
Unemployed	(r)			
Employed	1.55 (0.88–2.72)	.129		
Gender-affirming surgery				
Never	(r)			
Had surgery	1.38 (0.80–2.39)	.248		
Sources of hormone knowledge				
Hospital/clinic	(r)			
Internet	1.26 (0.67–2.39)	.468		
Social influencer /blogger	0.41 (0.14–1.15)	.090		
Friends or family	1.52 (0.54–4.28)	.426		
Advertisements	-			
Others	-			
Under medical supervision				
Yes	(r)			
No	1.78 (1.04–3.05)	.037*	1.21 (0.54–2.73)	.646

AOR = adjusted odds ratio; CI = confidence interval; OR = odds ratio; r = reference variable.

⁎ *P* < .05: significant differences from reference variable.

## DISCUSSION

The present cross-sectional observational study in a developing country reveals a high prevalence of GAHT use, up to 84% among TG. In addition, TW reported GAHT initiation at an earlier age than TM. TW commenced GAHT at age 18 years on average, while TM started at age 28. Previous data revealed the trend that transgender people in developing countries initiated GAHT at an earlier age than in developed countries. For example, studies from Thailand,^8^ and Laos,^13^ found that the median age of hormone initiation in TW was approximately 15–17 years, slightly lower than in the present study. However, studies from the United States^14^^,^^15^ and the Netherlands^16^ discovered the age of hormone initiation in TW around 27–33 years old, which was older than in our study. Moreover, the published studies found that TW in developed countries started GAHT at an older age than TM.^14^^,^^15^ In several developing countries, including Thailand, oral hormones could be purchased over the counter without a physician's prescription. In contrast, injectable hormones, such as injectable testosterone, are more difficult to access and require prescriptions. Thus, TW could self-prescribe hormones at an early age without medical supervision resulting in a GAHT initiation at a younger age than TM.

The previous data reported that approximately 10% of TW had medically unmonitored hormonal use.^15^^,^^17^ The present study shows that only 18% of TW received GAHT under medical advice, while most TM sought medical supervision. Since the age of gender identity realization was around 7–8 years old, more attention should be provided to TW, especially adolescents, regarding proper gender-affirming care. The primary source of information for GAHT in the present study is the internet, while hospitals/clinics are secondary. Evans et al. highlighted a need for reliable online content since the internet was the leading resource for transgender youth and caregivers.^18^ The content that was searched for online varied across the gender-affirming care, including discovering gender identity, retrieving knowledge, finding support networks, and seeking medical providers.^18^ Our study emphasized the necessity of trustworthy online content as a primary transgender health information resource. Medical society and professionals should provide accessible and understandable information for non-medical transgender people through online platforms.

The prevalence of using GAHT outside the reference regimen was 81% of TM and 67% of TW, according to the 2017 Endocrine Society Guidelines.^11^ The univariate analysis revealed that being TM is associated with a higher risk, approximately 2 times, of hormonal use outside the reference regimen. The result is noteworthy since most TM received gender-affirming treatment under medical supervision whereas there was easy access to unprescribed hormones among TW. However, being TM is associated with a higher risk of hormonal use outside the reference regimen. The authors realize that the GAHT is individualized. Therefore, the final hormone regimen may not be consistent with the recommended dosage in the standard guidelines but depends on the physician's decision. The breakdown of causes among TM with GAHT outside the reference regimen revealed two main reasons: higher doses per injection and less frequent intervals. This could result from the physician's decision to adjust medications yielding the final regimen outside the recommendation. Most data cited in the guidelines were based on the Caucasian population, while data in the Asian population is sparse. Two studies from Japan demonstrated the efficacy and safety of several testosterone regimens, including intramuscular testosterone enanthate 250 mg every 2 weeks and 250 mg every 3 weeks.^19^^,^^20^ These two testosterone regimens are also not in the recommended regimens from the Endocrine Society Guidelines. More data regarding the efficacy of various regimens apart from the recommended regimens are needed. Moreover, the results additionally raise the concern of whether physicians are knowledgeable in prescribing GAHT. Several data showed that a lack of knowledgeable medical providers was one of the main barriers to care for transgender individuals.^21^

Among TW, the main reasons for GAHT outside the reference regimen are using oral contraceptive pills and taking cyproterone acetate at 12.5 mg daily. All recommendations in feminizing hormones are against oral contraceptive pills since data showed a higher risk of venous thromboembolism and an inability to monitor estradiol levels.^11^ The reference regimen recommends cyproterone acetate 25–50 mg/d. Recent data revealed that low-dose cyproterone acetate (10–20 mg/d) suppressed testosterone level to a similar level compared with high-dose (50–100 mg/d).^22^ The present study revealed that low-dose cyproterone acetate is used in the real world, and more data is needed to prove its efficacy in testosterone suppression.

The feminizing hormone patterns depend on national availability. Most TW in the present study used oral estradiol and cyproterone acetate as an antiandrogen agent. This treatment pattern is similar to European countries where cyproterone acetate is the most commonly prescribed androgen-lowering medication.^23^ Spironolactone is commonly used in the United States, where cyproterone acetate is unavailable.^6^ Dosing several tablets per day and the potential side effect of hyperkalemia limit the utility. Only one TW reported using spironolactone in the present study. The National Health Service in the United Kingdom provides GnRH agonists for TW.^23^ GnRH agonists are not popular in Thailand due to their high cost. Surprisingly, data regarding feminizing hormones in Asian countries is scant. A study of 111 TW in Malaysia reported the common regimens were oral estrogen <4 mg/d, and up to 55% of TW used injectable estrogen. Half of them did not use antiandrogen, while only 30% used cyproterone acetate.^24^ Another study in 45 Vietnamese TW revealed that all used oral contraceptive pills with the highest dose of 15 pills per day.^25^ On the contrary, the masculinizing hormone pattern is similar across nations, including Asian countries, as intramuscular testosterone is commonly prescribed. A high dose of 250 mg intramuscular testosterone per injection was reported in Japanese studies.^19^^,^^20^^,^^26^ More data is needed to understand the pattern of GAHT in Asian countries.

Apart from being TM, other factors associated with a higher risk of hormonal use outside the reference regimen included age at the time of participating in the survey, age of hormone initiation, and receiving GAHT not under medical supervision. However, after multivariate analysis, only the area of residence outside Bangkok, which shows a trend from univariate analysis, is associated with higher risk. Most healthcare services for transgender people are located in the capital city or the main cities. Therefore, enhancing health literacy in GAHT among TG is vital, especially TG not living in the capital city.

Although the transgender community and transgender care have been expanded worldwide, several issues need to be addressed regarding health care policy for transgender people in Thailand. All costs involved in the gender-affirming treatment are self-pay since it is not subsidized by the government, including side effects from the treatment despite guidelines and data supporting the benefit of the therapy. There is no published study regarding the barrier to care among Thai transgender people. Anecdotally, the main barriers are similar to published data,^21^ including lack of education among providers, lack of access to care, social stigmatization, socioeconomic issues, and unsupported health system frameworks. Most academic hospitals in the public system provide transgender care. However, these centers are primarily located in the capital city, and accessibility remains a problem. The Thai Health Promotion Foundation, supported by the Thai government, has recently launched a project in 2021 to establish a transgender care clinic across all regions in Thailand. This project aims to close the gap of the lack of health care facilities providing transgender care and ultimately improve access to care.

The limitations of the present study should be addressed. First, given the nature of the cross-sectional observation study via questionnaire, causality could not be concluded, and results come with a recall bias. Second, this study exclusively included TG who have access to the internet. Although the internet is easily accessed nowadays, the study might have overlooked TG who do not engage in social media or do not use the internet regularly. Third, this study included a small sample size with a relatively young age. Therefore, the results do not represent the TG in an older age group. Expanding the study to cover more TG would represent the community's real-world practice of gender-affirming care.

## CONCLUSIONS

While the knowledge in gender-affirming care is expanding, this cross-sectional observational study in a developing country reveals a high prevalence of GAHT outside the reference regimen among TG. The only factor associated with hormonal use outside the reference regimen is living outside the capital city. Future research should focus on the efficacy and safety of various regimens apart from the recommended regimens in the guidelines. Moreover, future studies should be done to elucidate the reasons behind using GAHT outside the reference regimen. In addition, the capacity of medical providers in gender-affirming care and the barriers to care among transgender people should be evaluated. Enhancing health literacy in GAHT among TG is vital, especially TG not living in the capital city.

## STATEMENT OF AUTHORSHIP

Conceptualization: S.I., S.A., N.M., S.K.; data curation: S.I., S.A., N.M.; formal analysis: S.I., S.A., N.M.; methodology: S.I., S.A., N.M., S.K.; project administration: S.K.; resources: S.I., S.A., N.M.; software: S.I., S.A.; supervision: S.K; validation: S.K.; visualization: S.K.; writing – original draft: S.I., S.A., N.M.; writing – review: S.K.; editing: S.I., S.A., N.M., S.K.
